# The Association between Hepatic Encephalopathy and Diabetic Encephalopathy: The Brain-Liver Axis

**DOI:** 10.3390/ijms22010463

**Published:** 2021-01-05

**Authors:** So Yeong Cheon, Juhyun Song

**Affiliations:** 1 Department of Biotechnology, College of Biomedical & Health Science, Konkuk University, Chungju 27478, Korea; sycheon14@kku.ac.kr; 2 Department of Anatomy, Chonnam National University Medical School, Hwasun 58128, Jeollanam-do, Korea

**Keywords:** hepatic encephalopathy, diabetic encephalopathy, brain-liver axis, neurotransmitter, blood–brain barrier (BBB)

## Abstract

Hepatic encephalopathy (HE) is one of the main consequences of liver disease and is observed in severe liver failure and cirrhosis. Recent studies have provided significant evidence that HE shows several neurological symptoms including depressive mood, cognitive dysfunction, impaired circadian rhythm, and attention deficits as well as motor disturbance. Liver disease is also a risk factor for the development of diabetes mellitus. Diabetic encephalopathy (DE) is characterized by cognitive dysfunction and motor impairment. Recent research investigated the relationship between metabolic changes and the pathogenesis of neurological disease, indicating the importance between metabolic organs and the brain. Given that a diverse number of metabolites and changes in the brain contribute to neurologic dysfunction, HE and DE are emerging types of neurologic disease. Here, we review significant evidence of the association between HE and DE, and summarise the common risk factors. This review may provide promising therapeutic information and help to design a future metabolic organ-related study in relation to HE and DE.

## 1. Introduction

Liver failure is a major cause of morbidity and mortality worldwide [1], and is known to contribute to the onset and development of the pathogenesis of neurological diseases [2]. Encephalopathy is a general term for neurological dysfunction and can manifest in different ways [3]. Hepatic encephalopathy (HE) is a common consequence of late-stage liver disease that can result from multiple causes including liver failure, cirrhosis, and hepatitis [4]. HE has multiple known neurological and neuropsychiatric symptoms including mental disturbance, impaired circadian rhythm, and cognitive decline [5,6]. The long term potentiation (LTP) levels are reduced in the brain with HE, which influences motor function, attention, visual perception ability, synaptic transmission, learning and memory function, and cognition [7,8]. Several studies demonstrated that liver disease directly influences alteration of metabolites, such as ammonia, glucose, lactate, and glycine [9], and aggravates neuroinflammation and blood–brain barrier (BBB) breakdown [10,11]. Previous study mentioned that excessive ammonia level in HE is considered as the critical pathogenic factor involving in the progress of Alzheimer’s disease (AD) [12]. Elevated ammonia level leads to astrocyte and glia activation, and accelerates the deposition of amyloid beta proteins through abnormal lysosomal processing of amyloid beta precursor protein [12,13].

Diabetic encephalopathy (DE) is a chronic consequence of diabetes and displays cognitive and motor impairment [14]. Patients with DE show characteristic changes in the brain, ranging from the angiopathy of blood vessels, demyelination of cranial nerve, and neuronal changes that lead to cognitive decline. However, clear criteria for diagnosing DE are still lacking [15,16]. Patients with diabetes show a higher risk of dementia than do healthy individuals in clinical studies [17], and there is a considerable correlation between diabetes and the development of AD [18]. Furthermore, the prevalence of dementia and diabetes is simultaneously increasing [19]. Also, patients with type 2 diabetes showed the double risk of AD onset, and they showed amyloid beta deposit and brain insulin resistance compared to the normal subjects [20]. Therefore, some researchers addressed as type 3 diabetes based on these common pathological patterns between dementia and diabetes [19]. The onset and progression of diabetes-induced dementia, are considerably affected by metabolic pattern changes [21]. Diabetic animals exhibit BBB breakdown, neurodegeneration, and damaged cognitive function [22].

Liver disease is implicated in the development of diabetes and diabetes mellitus exacerbates brain damage in liver cirrhosis [23]. Clinically, patients with liver cirrhosis have altered peripheral glucose tolerance, insulin resistance, and inflammation similar to the patients with type 2 diabetes [24], and the combination of these diseases shows synergistic effect [25]. Diabetes is strongly related to fatty liver disease and then progresses to cirrhosis, which can result from insulin resistance and obesity [26]. In cirrhotic patients, diabetes increases the risk of HE, and patients with diabetes are at higher risk of developing cirrhosis and severe liver disease [25,27]. It is reported that diabetes develops as hepatogenous diabetes after liver disease (cirrhosis) onset [24]. Also, Mendelian randomization (MR) study proved that increased risk of diabetes is associated with genetic predisposition to elevated circulating aspartate aminotransferase (AST) and alanine aminotransferase (ALT), which are markers of liver function [28]. High concentration of these markers in circulation was detected in patients with liver disease [29]. Also, ammonia is known to increase in liver disease, but it has also been reported to increase in diabetes [27]. Although these two disorders might have different initiating factors, they share similar manifestations during disease progression [14,27] (Table 1).

Also, Metformin, one of the medications for treatment of type 2 diabetes, has shown protective effects against HE in diabetic cirrhotic patients [30].

Considering these correlations between liver and brain function, recent studies have focused on the brain-liver axis [31]. Diabetes and liver disease are metabolic disease. They generally show disrupted normal metabolism [32], therefore, these two diseases can be complications of each other. Although the link between liver failure and brain dysfunction is clear, the specific mechanism and related risk factors have not been fully understood until now. Here, we review recent evidence on the relationship between HE and DE. We summarise common causes and linking risk factors. In doing so, we highlight the need for future studies on the association between HE and DE.

## 2. Hepatic Encephalopathy 

The liver is considered as the largest metabolic organ and has important physiological functions, such as macronutrient metabolism, detoxification, hormone secretion, blood volume regulation, and digestion [33]. Liver cirrhosis has many causes including alcohol abuse and hepatitis C, and is characterized by the fibrous alteration of the liver [34]. One of the main consequences of liver cirrhosis is HE, occurring in approximately 30–45% of liver cirrhosis patients [35]. HE is associated to symptoms including anxiety, depressive symptoms, sleep disturbance, cognitive impairment, coma, and motor abnormalities and decline [5,6,36]. With increasing aging, it alters cognitive function in patients with HE [37]. Based on the West Haven Criteria, HE is subdivided into mild and severe HE. Covert/minimal HE (minimal or grade I) patients show neurocognitive impairment but have difficulty to be diagnosed clinically. However, covert/minimal HE can progress to overt HE (grade II-IV), which is a leading cause of hospitalizations [38,39].

HE shows neuropsychiatric symptoms by increasing brain edema and intracranial hypertension [40]. In chronic liver injury, the bile duct ligation (BDL) model demonstrates showing the pathology of chronic liver failure, including impaired liver function and fibrosis, that contributes to impaired brain functions and motor activity [41]. Abnormal liver function caused by hepatic damage results in multiple neurological symptoms including brain edema, changes to neuronal cell morphology, and neuron and glia cell function [42]. HE is the result of inappropriate hepatic detoxification that subsequently leads to excessive accumulation of toxic nitrogenous compounds in the body [36]. In HE, ammonia crosses the BBB and ammonia uptake is increased in the brain and cerebrospinal fluid (CSF) [43]. This hyperammonaemic condition triggers morphological alteration and dysfunction in glia and neuron, by inducing astrocyte swelling and brain edema [44]. Similarly, BDL model induces hyperammonaemia, subsequently leading to microglial activation, release of pro-inflammatory mediators, neuroinflammation, ultimately leading to neurological pathology [45]. Moreover, HE shows abnormal synthesis and secretion of neurotransmitters in the brain, which contribute to neurological pathology [46]. Previous literature highlighted that the increased level of glutamate in the brain is a critical sign of HE [47]. Also, energy metabolism including glucose and oxygen is changed in HE [48].

Therefore, liver disease significantly contributes to neurological pathologies in HE, including metabolic alterations, BBB breakdown, exacerbation of the inflammatory response, and cognitive and motor impairment.

## 3. Diabetic Encephalopathy

Diabetes is characterized by poor glycaemic control in the body; subsequently, these conditions can worsen the pathogenesis of many other neurological conditions. In particular, type 2 diabetic patients exhibit persistent hyperglycaemia, which causes distinct larger white matter lesion volume, total brain atrophy (lower gray and white matter volume), and cerebral infarction, resulting in cognitive impairment [49].

Liver is an important organ for glucose homeostasis. Liver cirrhosis can alter glucose metabolism and cause diabetes. Liver damage results in poor insulin clearance, which contributes to elevated systemic insulin and hyperinsulinaemia-induced insulin resistance [27]. Hyperglycaemia influences adhesion molecules in microvascular endothelial cells, and BBB integrity and permeability [50]. Similarly, patients with diabetes have impaired vasculature, more frequently develop cerebral infarction [49], and exhibit microvascular lesions and brain atrophy in the hippocampus and amygdala [18], which are important regions for learning and memory [18]. Patients with type 2 diabetes have some outcomes, such as abnormal brain structures and verbal memory impairment [51]. Previous literatures suggested that cognitive decline in diabetes is implicated in impaired or poor glucose tolerance, dysfunctional neurovascular coupling, and BBB disruption [52]. In addition, not only hippocampal synaptic plasticity and neuronal damage, but also metabolic and vascular alteration are responsible for DE and diabetes-associated dementia [53]. In diabetes, microglial activation and inflammatory response are observed in the brain [54].

On the basis of previous literatures, similar to HE, DE shows metabolic changes, impaired BBB, inflammation, and neurological dysfunction.

## 4. Common Risk Factors in Hepatic Encephalopathy and Diabetic Encephalopathy

### 4.1. Brain Structural Abnormalities Both in Hepatic Encephalopathy and Diabetic Encephalopathy 

The BBB is the barrier that protects the brain from various macromolecules in the blood circulation, and maintains the physiological homeostasis within the central nervous system (CNS) [55]. Many liver disease patients exhibit episodes of higher water content in the white matter and brain edema [56]. Aquaporin (AQP)-4, a bidirectional transmembrane water channel protein, is commonly found in astrocyte end-feet, which plays a regulatory role in brain water homeostasis [57]. AQP-4, which is related to BBB permeability and the formation of brain edema, is known to be dysregulated in both HE and liver disease [10,58]. In a post-mortem examination of the frontal cortex tissue from acute liver failure patients, increased levels of AQP-4 in the astrocytic end-feet were detected around the blood vessels [58]. In HE, the increase in AQP-4 was observed in brain regions, such as the cortex, striatum, cerebellum, and hippocampus, and the expression of tight junction proteins, including claudin-5, zona occluden-1 (ZO-1), and occludin, was decreased in the same brain regions [59]. In addition, diabetic patients show altered bile acid metabolism, the level of bile acid is higher than normal subjects, and increased bile acids are responsible for BBB opening [60]. Also, bile acid concentrations in the blood are significantly increased following liver injury [61]. Bile acids could affect BBB integrity by changing the tight junction structures, thereby leading to increased BBB permeability in a bile duct ligation HE animal model [62]. Finally, this elevated bile acid concentration in the blood stream triggers BBB disruption [61]. The increased bile acid level in the blood is closely associated with increased ammonia levels in the brain [61]. Excessive systemic ammonia, which can be absorbed by the brain, can cause cerebral edema [63].

In diabetes, BBB integrity is damaged, leading to an increase in BBB permeability [64]. Clinical studies showed diabetic individuals presented with impaired vasculature [49] and memory impairment [51]. Diabetic animals show decreased BBB integrity and enlarged gaps between tight junctions or loss of tight junction proteins, such as ZO-1 and occludin, finally resulting in BBB breakdown, which contributes to enhanced neurodegeneration and poor cognitive performance [22,65]. Impaired blood glucose management in diabetes affects the microvasculature, contributing to BBB impairment and brain edema [66]. Under hyperglycaemic conditions, AQP-4 is involved in BBB breakdown and brain edema [67]. An in vitro study demonstrated that silencing *AQP-4* gene expression in astrocytes reduces water permeability [68].

Glucose and lactate secreted by astrocytes are the main bioenergetic sources for neurons [69]. Several studies found an increased lactate level and the formation of brain edema in HE [63,70]. Elevated lactate levels in the brain could induce brain edema in liver disease [71]. Increased lactate is closely associated with insulin resistance and higher incidence of diabetes, and type 1 and type 2 diabetic models have shown that hyperglycaemia is linked to enhanced BBB permeability [72].

Given these findings, changes in brain structure, such as BBB breakdown and brain edema, lead to disrupting brain function, and are common risk factors both in HE and DE.

### 4.2. Aberrant Glia and Neuroinflammation Both in Hepatic Encephalopathy and Diabetic Encephalopathy 

**Figure 1:**
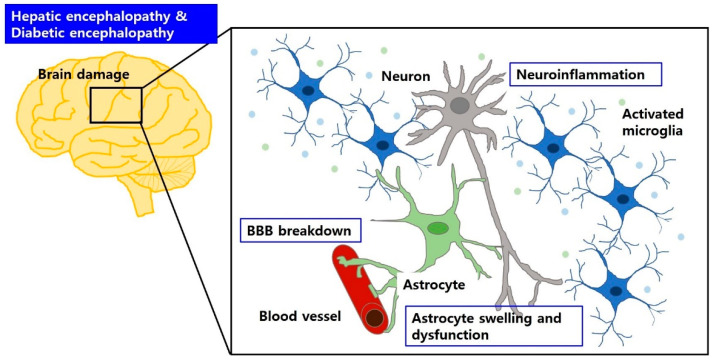
BBB breakdown and brain edema may aggravate brain dysfunction in HE and DE. Circulating factors can cause BBB breakdown, accompanied by astrocyte swelling and dysfunction, and glial activation. These can induce neuroinflammation, which is responsible for neuronal cell damage in HE and DE.

Astrocytes are a sub-type of glia in the CNS that provide metabolic support to neurons and are essential for maintaining the BBB structure and function [73]. Glial fibrillary acidic protein (GFAP), an intermediate filament found in astrocytes, helps to maintain the structural stability of the astrocyte [42]. A previous study showed that the reduction in GFAP after acute liver failure was involved in astrocyte swelling [58], and significant decrease or increase in GFAP was also observed in the brain regions, such as hippocampus and cerebral cortex, from diabetic animals [74]. Also, diabetic condition leads to astrocyte dysfunction and neuroinflammation [72] (Figure 1).

It has been known that astrocytes are responsible for ammonia detoxification and glutamate homeostasis in the brain [75]. In astrocytes of the brain, glutamate synthesis is directly involved in glutamine metabolism, and the synthesis of glutamine occurs in astrocytes [76]. In a healthy body, ammonia that does not enter the urea cycle should be metabolised by glutamine synthetase in the liver to generate glutamine from glutamate [77]. Also, ammonia can cross the BBB through the processes of astrocytes and could be metabolised with glutamate through the action of glutamine synthetase in astrocytes in the brain [78]. However, in hyperammonaemia, astrocytes undergo functional changes that may reflect the altered levels of glutamine synthetase [78]. Therefore, in HE, elevated ammonia concentrations are related to the increase in glutamine production in astrocytes, and the accumulation of osmotic active glutamine induces osmotic stress within astrocytes [79]. Clinical study reported that high levels of glutamine were detected in the brain as well as CSF [80]. Other study suggested that the activity of glutamine synthetase was markedly reduced in the hyperammonaemic brain regions, such as the hippocampus, cerebral cortex, and cerebellum, in a model of chronic liver failure [81]. Under diabetic conditions, brain glutamate levels are increased, compared with healthy controls [82]. Hyperglycaemia can change astrocyte morphology and metabolism. In particular, high level of lactate production in astrocytes is associated with hyperglycaemia [83].

Both HE and DE may be characterized by primary peripheral/systemic inflammation, which can influence cerebral inflammation [84,85]. Microglia control the immune and inflammatory responses in the CNS [86]. Liver disease and diabetes influence the functional change and polarization of microglia in the brain [10,54]. Previous studies have shown that microglial activation is observed in HE and diabetes, and the activated microglia produce various inflammatory cytokines [54,87]. These cytokines can induce axonal degeneration and apoptosis [88].

Researchers have proven that HE commonly exhibits high levels of secretion of pro-inflammatory chemokines, including interleukin-6 (IL-6), interleukin-1β (IL-1β), tumor necrosis factor-α (TNF-α), and chemokine monocyte chemoattractant protein-1, and induces more pro-inflammatory microglia (M1 phenotype) than anti-inflammatory (M2 phenotype) [89]. Also, chronic diabetes induces pro-inflammatory cytokines including IL-1β and interferon-γ (IFN-γ), and diabetic animals show increased pro-inflammatory TNF-α and decreased anti-inflammatory IL-10 [54,90]. Although the cytokine profile differs between HE and DE, they share the same cytokine profile, including IL-1β and TNF-α [54,89,90]. Therefore, targeting these cytokines may be helpful to investigate therapeutic tools for HE and DE.

Neuroinflammation due to the BBB breakdown, and astroglial and microglial activation contributes to cognitive and motor dysfunction [88]. In a clinical study, the severity of cognitive dysfunction depends on the levels of circulating ammonia and pro-inflammatory cytokines, such as IL-6 and IL-18. The interplay between hyperammonaemia and inflammation is involved in cognitive impairment in HE patients [91]. Microglial activation and neuroinflammation are observed in patients with HE by using position emission tomography. In particular, these are detected in the anterior cingulate cortex, which is involved in attention [92]. Suppression of inflammation by anti-inflammatory agents, such as ibuprofen, restores impaired memory ability and motor function by inhibiting microglial activation and neuroinflammation in BDL animal models of HE [45]. Also, anti-inflammatory agents result in reduced incidence and the progression of diabetes [93]. In non-alcoholic fatty liver disease (NAFLD), inflammatory response in the liver is elevated by hyperglycaemia, and involved in disease progression [94]. Also, hyperglycaemia-driven neuroinflammation is related to memory loss in type 1 and 2 diabetes [72].

Based on previous data, astrocyte dysfunction, microglia activation, and its pro-inflammatory cytokine release are implicated in neurological dysfunction under HE and DE.

### 4.3. Imbalance of Neurotransmission Both in Hepatic Encephalopathy and Diabetic Encephalopathy

Adults with HE show abnormal neurotransmitters production [95], leading to behavioural disturbances and various neuropathologies, including altered neuronal circuits [96]. Motor disturbances, such as slow movement and simultaneous memory loss, are routinely found in HE patients [70]. In diabetes, abnormal production of neurotransmitters is observed in the brain, which can cause low cognitive function and depression [97].

One study demonstrated that LTP in the hippocampus, which is involved in memory function and homeostatic modulation of neurotransmitter, was markedly decreased in the brain from HE patients relative to that in the healthy brain [98]. Morphological changes, such as size and structure of neuronal cells, are observed in diabetic animal model. They exhibit impaired learning and memory [99].

Some studies have reported that an increased extracellular GABA neurotransmitter in the brain regions, such as the cerebellum, is directly linked to the decrease in motor activity, and is related to impaired neural circuits (basal ganglia-thalamus-cortex circuits) and increased cognitive decline under HE or hyperammonaemic conditions [100,101]. Furthermore, GABA receptors and transporters are expressed at increased levels in the HE brain, and ultimately influence poor motor coordination [100]. In diabetes, the level of GABA is increased in the hippocampus [97]. Patients with type 2 diabetes show high levels of GABA in the blood, leading to poor cognitive performance [102].

In the CNS, serotonin homeostasis contributes to various pathological mechanisms including the immune system and emotional disturbance [103]. Dopamine is important in motor function, motivational function, and memory consolidation [104]. Impaired serotonin homeostasis and uptake are linked to liver cirrhosis [105]. A previous study demonstrated that serotonin level was increased in the CSF and serum in HE patients [106]. Other study has suggested that abnormal increased dopamine levels are related to the induction of HE, and the abnormal elevated dopamine level in the HE brain is associated with learning and memory dysfunction [107].

However, a previous study reported the opposite: decreased levels of dopamine and serotonin in the brain [108]. In this study, reduced dopaminergic and serotonergic system activity in brain regions, such as the cortex, hippocampus, striatum, and cerebellum, led to neuronal degeneration, impaired learning and memory, anxiety-like symptoms, and motor dysfunction in HE patients [108]. In diabetes model, reduced level of dopaminergic neurotransmission in the amygdala results in impaired social recognition memory [109]. Also, diabetic animal models (insulin receptor deficiency) show reduced dopamine release in the striatum, and they display anxiety- and depression-like behaviors [110].

The noradrenaline (or norepinephrine) system also plays an essential role in prefrontal cortical and hippocampal functions; therefore, it controls cognitive function [111]. However, a rat model of HE showed elevated metabolic noradrenergic activity in the brain, accompanied by a loss of memory and mental disturbance [112]. Obese diabetic mice show different concentration of norepinephrine, depending on their aging. Mice at 4 weeks of age exhibit increased level of norepinephrine in the cortex, and mice at 8 weeks of age show elevated levels of norepinephrine in the cortex, septum, hypothalamus, and medulla, compared to age-matched controls, implying neuronal degeneration [113].

Generally, glutamate is necessary for the regulation of cortical functions including learning and memory [114]. A dysregulated glutamate system contributes to neurodegenerative and neuropsychiatric diseases [114]. As mentioned above, liver damage induces abnormalities in the glutamatergic system in the brain and leads to behavioural alterations [115]. Elevated extracellular glutamate levels lead to motor dysfunction in hyperammonaemic conditions and in HE [116]. Elevated glutamate levels are detected in the hippocampus and increased levels of glutamine are observed in the prefrontal cortex and hippocampus [97]. Patients with type 1 diabetes have low cognitive function and mild depression due to high glutamate levels in the prefrontal cortex [117].

Choline is the precursor for another neurotransmitter, acetylcholine, that is directly involved in memory function [118]. Cholinergic stimulation helps improve declining memory [118], but decreased choline transport across the BBB is observed in a diabetic rat model [119]. HE patients show decreased concentrations of choline in the brain [120].

Also, liver disease and diabetes share genetic variances. For instance, HE patients with cirrhosis showed genetic polymorphism of glutaminase gene [121]. Genetic risk factors for liver disease (NAFLD) were associated with lipid and glucose metabolism, including peroxisome proliferator-activated receptor-alpha (*PPARA*), *PPARG*, *LIPIN1*, and insulin receptor substrate 1 (*IRS1*) [122]. In diabetes, the genes associated with lipid and glucose metabolism, such as *PPARγ*, *IRS1* and *IRS2* were genetic risk factors [123], and hepatic glutaminase mRNA was abundant during diabetes [124]. Therefore, dysregulated lipid or glucose metabolism might be the risk factor for HE and DE.

**Figure 2:**
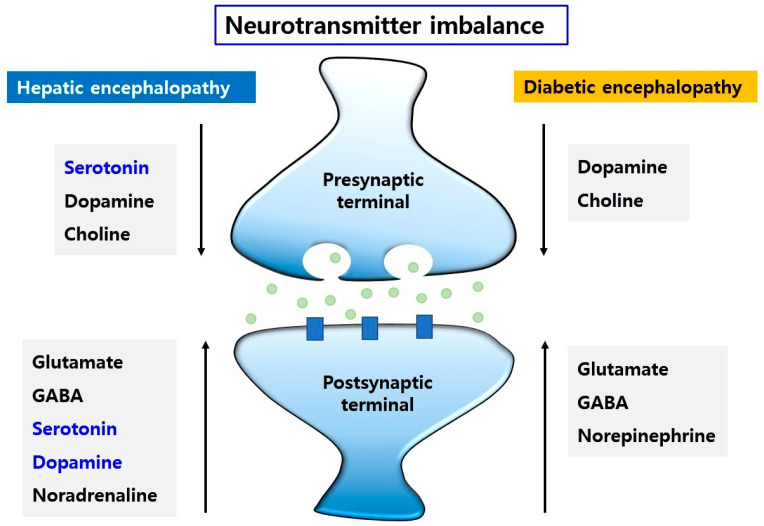
The neurotransmitter imbalance in HE and DE. The changes in neurotransmitters, such as glutamate, GABA, serotonin, dopamine, and noradrenaline, may lead to motor dysfunction, learning and memory dysfunction, and emotional disturbance. The levels of dopamine and choline are decreased and the levels of glutamate, GABA, and norepinephrine are increased in HE and DE. However, Dopamine and serotonin levels are elevated in HE. Conversely, serotonin level is reduced in HE.

Collectively, changes in the levels of some neurotransmitters, in brains of HE and DE (Figure 2) contribute to neurological dysfunction with emotional changes, motor disturbances, and memory loss.

### 4.4. Insulin Resistance and Impaired Glucose Metabolism Both in Hepatic Encephalopathy and Diabetic Encephalopathy

In the CNS, glucose metabolism and insulin function are very important for neuronal functions and cerebral networks [125], suggesting that the brain is an insulin-sensitive organ [126].

Insulin regulates glucose utilisation in the CNS, and ultimately controls cognitive function, neuronal cell growth, and energy homeostasis in the brain [127,128]. Insulin binds to specific receptors located within many brain regions, including the hypothalamus, hippocampus, and cerebral cortex, and acts as a regulator of cellular function [128]. Appropriate insulin action through insulin receptors in the brain contributes to the maintenance of neuronal cell survival, synaptic plasticity, and memory function [129]. Therefore, insulin resistance may be a critical index for the measurement of cognitive decline and diagnosis of dementia [129].

**Figure 3:**
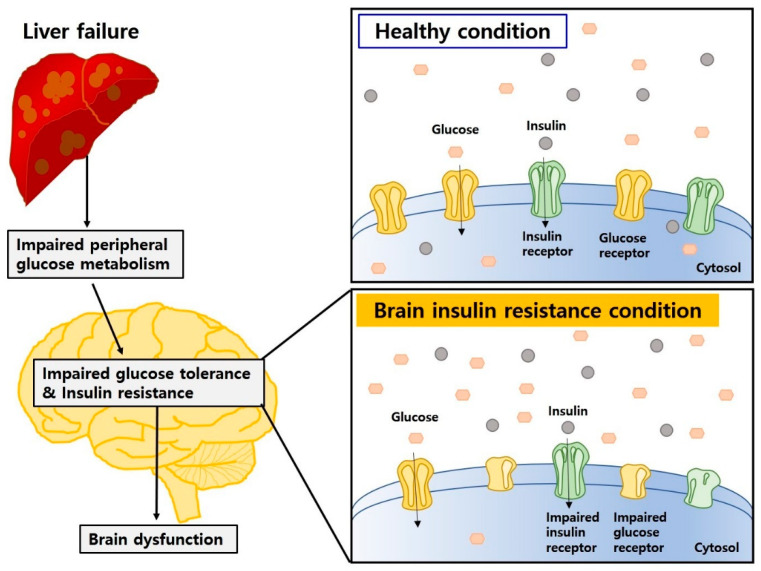
Insulin resistance and impaired glucose metabolism may aggravate brain dysfunction in HE and DE. Peripheral insulin and glucose metabolism can influence brain homeostasis, and impaired glucose tolerance and insulin resistance can cause brain dysfunction in HE and DE.

Impaired insulin action in the CNS influences the onset and progression of neurodegenerative diseases [130]. Previous study has demonstrated that insulin resistance and impaired glucose metabolism resulted in cognitive decline and aggravated the progression of dementia [131] (Figure 3). 

The liver is the major organ that regulates general glucose metabolism, including the balance of glucose uptake, glycogenesis, and gluconeogenesis [132]. Previous study highlighted that patients with liver disease have similar insulin resistance to type 2 diabetes patients [24]. Also, type 2 diabetic mice share the same features in chronic liver disease, NAFLD, such as insulin resistance and altered glycaemic homeostasis, which are also main characteristics in type 2 diabetes [133]. Moreover, diabetes worsens the progression of liver fibrosis [134] and elevates the risk of HE in patients with cirrhosis [25], indicating a close link between HE and diabetes.

Normally, glucose can cross the BBB through transporter proteins, such as sodium-independent glucose transporters (GLUT) and sodium-dependent glucose co-transporters (SGLT) [135]. However, glucose transporter activity in the BBB was downregulated in an experimental model of diabetes [136]. Transcription of the glucose transporter subunit, GLUT-1, was reduced in the BBB but GLUT-1 protein levels showed no change in diabetic mice [137], indicating an abnormal glucose level in the brain. In acute liver failure, increased levels of GLUT1 and glucose uptake are observed in the brain [138]. Also, the inhibition of SGLT1 or SGLT2 displays glucose lowering effect and improves glucose homeostasis in diabetes [139]. Also, SGLT1/SGLT2 inhibition prevents NAFLD progression and reduces glucose production in hepatocyte of type 2 diabetic animals [133]. In addition, patients with cirrhosis showed impaired brain glucose metabolism, even though they had no overt HE [140].

Considering previous studies, insulin resistance and impaired glucose metabolism are associated with neurologic dysfunction and cognitive decline in both HE and DE.

## 5. Conclusions 

As mentioned above, there are many common risk factors between HE and DE. Many researchers have shown the treatments for these pathologies including nutritional therapy for the regulation of nitrogen metabolism, probiotics therapy for reduction of bacterial urease activity in gut [141], antibiotics therapy such as neomycin [142], L-Ornithine-l-aspartate supplement therapy for promotion of urea cycle [143], and finally liver transplantation [144]. However, each clinical approach has some limitations. For treatment of these pathologies, we need to find more appropriated clinical approach through future studies.

Here, we reviewed common risk factors in both HE and DE. We highlighted the four crucial points in common between HE and DE based on previously published evidence.

First, the BBB is an important barrier that regulates metabolite movement between the systemic circulation and the CNS. Damage to the BBB in HE and in DE can alter the metabolic system and negatively influence vascular homeostasis both in metabolic organs and in the brain. Preventing BBB breakdown in HE and DE may be important in preventing neuropathologies.

Secondly, abnormal glial function and inflammation are influenced by metabolic factor changes, and subsequently damages both the liver and the brain. The modulation of microglia and astrocyte function may be the key to attenuating neuropathology in HE and DE.

Thirdly, neurotransmitters, including serotonin, GABA, dopamine, and glutamine, regulate behavioural patterns and mood, memory function, sleep patterns, and attention. The regulation of neurotransmitter imbalances in HE and DE may be the cardinal issue to solve many neuropathologies associated with HE and DE.

Finally, insulin resistance regulates neuronal cell function and glial activation in the brain, as well as metabolic organ function. Restoring insulin sensitivity in HE and DE may solve multiple neuropathologies.

We have highlighted that metabolic problems could influence both organ functions, such as liver and brain functions. Studies on the relationship between common risk factors, observed in liver disease and diabetes, and neurological pathologies are necessary to find therapeutic solutions. Thus, we suggest that common risk factors linking HE and DE may provide promising information that could address multiple pathologies.

## Figures and Tables

**Table 1:** Similarities and differences between hepatic encephalopathy and diabetic encephalopathy.

	Similarities between Hepatic Encephalopathy and Diabetic Encephalopathy	Differences between Hepatic Encephalopathy and Diabetic Encephalopathy
**Brain vasculature**	Increased BBB permeability BBB breakdown Increased level of water channel molecule, AQP-4 Decreased levels of tight junction molecules, such as ZO-1 and occluding Changes in BBB integrity and permeability by increased bile acid	Brain edema induced by hyperammonaemia in HE
**Glial cells**	Abnormal astrocyte function in glutamine-glutamate homeostasis Astrocyte swelling Microglial activation and secretion of inflammatory cytokines	Levels of GFAP in HE and DE Astrocyte dysfunction in ammonia detoxification in HE Hyperammonaemia-driven neuroinflammation in HE
**Neurotransmitters**	Impairment of neuronal function Changes in GABA, glutamate, choline levels	Changes in serotonin and dopamine levels in HE and DE
**Glucose metabolism**	Insulin resistance Impairment of glucose metabolism, including glucose uptake Protective effect after inhibition of SGLT1 and/or SGLT2	The level of GLUT1 in HE and DE
**Cognitive function**	Learning and memory impairment Disrupted attention Neuropsychological dysfunction (Anxiety and depression)	Progressive and mild cognitive dysfunction in DE Dynamic and reversible cognitive dysfunction in HE
**etc.**		Bacterial infection (Hepatitis) in HE Pancreatic beta cell loss in DE

## List of Abbreviations

Hepatic encephalopathy (HE); Central nervous system (CNS); Long-term potentiation (LTP); Reactive oxygen species (ROS); Blood–brain barrier (BBB); Bile duct ligation (BDL); Zona occluden-1 (ZO-1); Alzheimer’s disease (AD); Parkinson’s disease (PD); Aquaporin (AQP); Glucose transporter (GLUT); Sodium-dependent glucose co-transporter (SGLT); Glial fibrillary acidic protein (GFAP); Cerebrospinal fluid (CSF); γ-aminobutyric acid (GABA); Interleukin-6 (IL-6); Interleukin-1beta (IL-1β); Tumor necrosis factor-α (TNF-α).

## References

[1] GBD 2017 Cirrhosis Collaborators (2020). The global, regional, and national burden of cirrhosis by cause in 195 countries and territories, 1990-2017: A systematic analysis for the Global Burden of Disease Study 2017. Lancet Gastroenterol. Hepatol..

[2] Nardelli S., Allampati S., Riggio O., Mullen K.D., Prakash R., Gioia S., Unser A., White M.B., Fagan A.C., Wade J.B. (2017). Hepatic Encephalopathy Is Associated with Persistent Learning Impairments Despite Adequate Medical Treatment: A Multicenter, International Study. Dig. Dis. Sci..

[3] Jones E.A., Weissenborn K. (1997). Neurology and the liver. J. Neurol. Neurosurg. Psychiatry.

[4] Butterworth R.F., Norenberg M.D., Felipo V., Ferenci P., Albrecht J., Blei A.T., Members of the ISHEN Commission on Experimental Models of HE (2009). Experimental models of hepatic encephalopathy: ISHEN guidelines. Liver Int..

[5] Martino M.E., Fernandez-Lorente J., Romero-Vives M., Barcena R., Gaztelu J.M. (2014). Brain oscillatory activity during sleep shows unknown dysfunctions in early encephalopathy. J. Physiol. Biochem..

[6] Weissenborn K., Bokemeyer M., Krause J., Ennen J., Ahl B. (2005). Neurological and neuropsychiatric syndromes associated with liver disease. AIDS.

[7] Nardone R., Taylor A.C., Holler Y., Brigo F., Lochner P., Trinka E. (2016). Minimal hepatic encephalopathy: A review. Neurosci. Res..

[8] Wen S., Schroeter A., Klocker N. (2013). Synaptic plasticity in hepatic encephalopathy—A molecular perspective. Arch. Biochem. Biophys..

[9] Weiss N., Barbier Saint Hilaire P., Colsch B., Isnard F., Attala S., Schaefer A., Amador M.D., Rudler M., Lamari F., Sedel F. (2016). Cerebrospinal fluid metabolomics highlights dysregulation of energy metabolism in overt hepatic encephalopathy. J. Hepatol..

[10] Jaeger V., DeMorrow S., McMillin M. (2019). The Direct Contribution of Astrocytes and Microglia to the Pathogenesis of Hepatic Encephalopathy. J. Clin. Transl. Hepatol..

[11] Shawcross D.L., Shabbir S.S., Taylor N.J., Hughes R.D. (2010). Ammonia and the neutrophil in the pathogenesis of hepatic encephalopathy in cirrhosis. Hepatology.

[12] Seiler N. (2002). Ammonia and Alzheimer’s disease. Neurochem. Int..

[13] Agarwal A.N., Mais D.D. (2019). Sensitivity and Specificity of Alzheimer Type II Astrocytes in Hepatic Encephalopathy. Arch. Pathol. Lab. Med..

[14] Diaz-Gerevini G.T., Dain A., Pasqualini M.E., Lopez C.B., Eynard A.R., Repossi G. (2019). Diabetic encephalopathy: Beneficial effects of supplementation with fatty acids omega3 and nordihydroguaiaretic acid in a spontaneous diabetes rat model. Lipids Health Dis..

[15] Mijnhout G.S., Scheltens P., Diamant M., Biessels G.J., Wessels A.M., Simsek S., Snoek F.J., Heine R.J. (2006). Diabetic encephalopathy: A concept in need of a definition. Diabetologia.

[16] Wang C., Li J., Zhao S., Huang L. (2020). Diabetic encephalopathy causes the imbalance of neural activities between hippocampal glutamatergic neurons and GABAergic neurons in mice. Brain Res..

[17] Gudala K., Bansal D., Schifano F., Bhansali A. (2013). Diabetes mellitus and risk of dementia: A meta-analysis of prospective observational studies. J. Diabetes Investig..

[18] Cheng G., Huang C., Deng H., Wang H. (2012). Diabetes as a risk factor for dementia and mild cognitive impairment: A meta-analysis of longitudinal studies. Intern. Med. J..

[19] Biessels G.J., Strachan M.W., Visseren F.L., Kappelle L.J., Whitmer R.A. (2014). Dementia and cognitive decline in type 2 diabetes and prediabetic stages: Towards targeted interventions. Lancet Diabetes Endocrinol..

[20] Kroner Z. (2009). The relationship between Alzheimer’s disease and diabetes: Type 3 diabetes?. Altern. Med. Rev..

[21] Hanyu H. (2019). Diabetes-Related Dementia. Adv. Exp. Med. Biol..

[22] Takechi R., Lam V., Brook E., Giles C., Fimognari N., Mooranian A., Al-Salami H., Coulson S.H., Nesbit M., Mamo J.C.L. (2017). Blood-Brain Barrier Dysfunction Precedes Cognitive Decline and Neurodegeneration in Diabetic Insulin Resistant Mouse Model: An Implication for Causal Link. Front. Aging Neurosci..

[23] Wang Y.F., Kong X., Lu G.M., Zhang L.J. (2017). Diabetes Mellitus is Associated with More Severe Brain Spontaneous Activity Impairment and Gray Matter Loss in Patients with Cirrhosis. Sci. Rep..

[24] Garcia-Compean D., Jaquez-Quintana J.O., Gonzalez-Gonzalez J.A., Maldonado-Garza H. (2009). Liver cirrhosis and diabetes: Risk factors, pathophysiology, clinical implications and management. World J. Gastroenterol..

[25] Jepsen P., Watson H., Andersen P.K., Vilstrup H. (2015). Diabetes as a risk factor for hepatic encephalopathy in cirrhosis patients. J. Hepatol..

[26] Bhatt H.B., Smith R.J. (2015). Fatty liver disease in diabetes mellitus. Hepatobiliary Surg. Nutr..

[27] Elkrief L., Rautou P.E., Sarin S., Valla D., Paradis V., Moreau R. (2016). Diabetes mellitus in patients with cirrhosis: Clinical implications and management. Liver Int..

[28] De Silva N.M.G., Borges M.C., Hingorani A.D., Engmann J., Shah T., Zhang X., Luan J., Langenberg C., Wong A., Kuh D. (2019). Liver Function and Risk of Type 2 Diabetes: Bidirectional Mendelian Randomization Study. Diabetes.

[29] Sookoian S., Castano G.O., Scian R., Fernandez Gianotti T., Dopazo H., Rohr C., Gaj G., San Martino J., Sevic I., Flichman D. (2016). Serum aminotransferases in nonalcoholic fatty liver disease are a signature of liver metabolic perturbations at the amino acid and Krebs cycle level. Am. J. Clin. Nutr..

[30] Ampuero J., Ranchal I., Nunez D., Diaz-Herrero Mdel M., Maraver M., del Campo J.A., Rojas A., Camacho I., Figueruela B., Bautista J.D. (2012). Metformin inhibits glutaminase activity and protects against hepatic encephalopathy. PLoS ONE.

[31] Yao Y., Zhang W., Ming R., Deng Q., Zuo A., Zhang S., Ying Y., Zhao Y., Ma J. (2020). Noninvasive 40-Hz Light Flicker Rescues Circadian Behavior and Abnormal Lipid Metabolism Induced by Acute Ethanol Exposure via Improving SIRT1 and the Circadian Clock in the Liver-Brain Axis. Front. Pharmacol..

[32] Watanabe S., Yaginuma R., Ikejima K., Miyazaki A. (2008). Liver diseases and metabolic syndrome. J. Gastroenterol..

[33] Trefts E., Gannon M., Wasserman D.H. (2017). The liver. Curr. Biol..

[34] Schuppan D., Afdhal N.H. (2008). Liver cirrhosis. Lancet.

[35] Khungar V., Poordad F. (2012). Hepatic encephalopathy. Clin. Liver Dis..

[36] Suraweera D., Sundaram V., Saab S. (2016). Evaluation and Management of Hepatic Encephalopathy: Current Status and Future Directions. Gut Liver.

[37] Liu R., Ahluwalia V., Kang J.D., Ghosh S.S., Zhou H., Li Y., Zhao D., Gurley E., Li X., White M.B. (2019). Effect of Increasing Age on Brain Dysfunction in Cirrhosis. Hepatol. Commun..

[38] Bajaj J.S. (2008). Management options for minimal hepatic encephalopathy. Expert Rev. Gastroenterol. Hepatol..

[39] Ferenci P., Lockwood A., Mullen K., Tarter R., Weissenborn K., Blei A.T. (2002). Hepatic encephalopathy--definition, nomenclature, diagnosis, and quantification: Final report of the working party at the 11th World Congresses of Gastroenterology, Vienna, 1998. Hepatology.

[40] Lemberg A., Fernandez M.A. (2009). Hepatic encephalopathy, ammonia, glutamate, glutamine and oxidative stress. Ann. Hepatol..

[41] Hambuchen M.D., Berquist M.D., Simecka C.M., McGill M.R., Gunnell M.G., Hendrickson H.P., Owens S.M. (2019). Effect of Bile Duct Ligation-induced Liver Dysfunction on Methamphetamine Pharmacokinetics and Locomotor Activity in Rats. J. Pharm. Pharm. Sci..

[42] Butterworth R.F. (2015). Pathogenesis of hepatic encephalopathy and brain edema in acute liver failure. J. Clin. Exp. Hepatol..

[43] Sorensen M. (2013). Update on cerebral uptake of blood ammonia. Metab. Brain Dis..

[44] Sinke A.P., Jayakumar A.R., Panickar K.S., Moriyama M., Reddy P.V., Norenberg M.D. (2008). NFkappaB in the mechanism of ammonia-induced astrocyte swelling in culture. J. Neurochem..

[45] Rodrigo R., Cauli O., Gomez-Pinedo U., Agusti A., Hernandez-Rabaza V., Garcia-Verdugo J.M., Felipo V. (2010). Hyperammonemia induces neuroinflammation that contributes to cognitive impairment in rats with hepatic encephalopathy. Gastroenterology.

[46] Vaquero J., Butterworth R.F. (2007). Mechanisms of brain edema in acute liver failure and impact of novel therapeutic interventions. Neurol. Res..

[47] Savlan I., Liakina V., Valantinas J. (2014). Concise review of current concepts on nomenclature and pathophysiology of hepatic encephalopathy. Medicina (Kaunas).

[48] Jalan R., Hayes P.C. (1997). Hepatic encephalopathy and ascites. Lancet.

[49] Saczynski J.S., Siggurdsson S., Jonsson P.V., Eiriksdottir G., Olafsdottir E., Kjartansson O., Harris T.B., van Buchem M.A., Gudnason V., Launer L.J. (2009). Glycemic status and brain injury in older individuals: The age gene/environment susceptibility-Reykjavik study. Diabetes Care.

[50] Allen C.L., Bayraktutan U. (2009). Antioxidants attenuate hyperglycaemia-mediated brain endothelial cell dysfunction and blood-brain barrier hyperpermeability. Diabetes Obes. Metab..

[51] van Gemert T., Wolwer W., Weber K.S., Hoyer A., Strassburger K., Bohnau N.T., Bruggen M.A., Ovelgonne K., Gossmann E.M., Burkart V. (2018). Cognitive Function Is Impaired in Patients with Recently Diagnosed Type 2 Diabetes, but Not Type 1 Diabetes. J. Diabetes Res..

[52] Mogi M., Horiuchi M. (2011). Neurovascular coupling in cognitive impairment associated with diabetes mellitus. Circ. J..

[53] Gispen W.H., Biessels G.J. (2000). Cognition and synaptic plasticity in diabetes mellitus. Trends Neurosci..

[54] Hwang I.K., Choi J.H., Nam S.M., Park O.K., Yoo D.Y., Kim W., Yi S.S., Won M.H., Seong J.K., Yoon Y.S. (2014). Activation of microglia and induction of pro-inflammatory cytokines in the hippocampus of type 2 diabetic rats. Neurol. Res..

[55] Bernacki J., Dobrowolska A., Nierwinska K., Malecki A. (2008). Physiology and pharmacological role of the blood-brain barrier. Pharmacol. Rep..

[56] Winterdahl M., Abbas Z., Noer O., Thomsen K.L., Gras V., Nahimi A., Vilstrup H., Shah N.J., Dam G. (2019). Cerebral water content mapping in cirrhosis patients with and without manifest HE. Metab. Brain Dis..

[57] Wright G., Soper R., Brooks H.F., Stadlbauer V., Vairappan B., Davies N.A., Andreola F., Hodges S., Moss R.F., Davies D.C. (2010). Role of aquaporin-4 in the development of brain oedema in liver failure. J. Hepatol..

[58] Thumburu K.K., Dhiman R.K., Vasishta R.K., Chakraborti A., Butterworth R.F., Beauchesne E., Desjardins P., Goyal S., Sharma N., Duseja A. (2014). Expression of astrocytic genes coding for proteins implicated in neural excitation and brain edema is altered after acute liver failure. J. Neurochem..

[59] Dhanda S., Sandhir R. (2018). Blood-Brain Barrier Permeability Is Exacerbated in Experimental Model of Hepatic Encephalopathy via MMP-9 Activation and Downregulation of Tight Junction Proteins. Mol. Neurobiol..

[60] Gonzalez-Regueiro J.A., Moreno-Castaneda L., Uribe M., Chavez-Tapia N.C. (2017). The Role of Bile Acids in Glucose Metabolism and Their Relation with Diabetes. Ann. Hepatol..

[61] Xie G., Wang X., Jiang R., Zhao A., Yan J., Zheng X., Huang F., Liu X., Panee J., Rajani C. (2018). Dysregulated bile acid signaling contributes to the neurological impairment in murine models of acute and chronic liver failure. EBioMedicine.

[62] Quinn M., McMillin M., Galindo C., Frampton G., Pae H.Y., DeMorrow S. (2014). Bile acids permeabilize the blood brain barrier after bile duct ligation in rats via Rac1-dependent mechanisms. Dig. Liver Dis..

[63] Haussinger D., Kircheis G., Fischer R., Schliess F., vom Dahl S. (2000). Hepatic encephalopathy in chronic liver disease: A clinical manifestation of astrocyte swelling and low-grade cerebral edema?. J. Hepatol..

[64] Acharya N.K., Levin E.C., Clifford P.M., Han M., Tourtellotte R., Chamberlain D., Pollaro M., Coretti N.J., Kosciuk M.C., Nagele E.P. (2013). Diabetes and hypercholesterolemia increase blood-brain barrier permeability and brain amyloid deposition: Beneficial effects of the LpPLA2 inhibitor darapladib. J. Alzheimers Dis..

[65] Geng J., Wang L., Zhang L., Qin C., Song Y., Ma Y., Chen Y., Chen S., Wang Y., Zhang Z. (2018). Blood-Brain Barrier Disruption Induced Cognitive Impairment Is Associated With Increase of Inflammatory Cytokine. Front. Aging Neurosci..

[66] Muresanu D.F., Sharma A., Sharma H.S. (2010). Diabetes aggravates heat stress-induced blood-brain barrier breakdown, reduction in cerebral blood flow, edema formation, and brain pathology: Possible neuroprotection with growth hormone. Ann. N. Y. Acad. Sci..

[67] Deng J., Zhao F., Yu X., Zhao Y., Li D., Shi H., Sun Y. (2014). Expression of aquaporin 4 and breakdown of the blood-brain barrier after hypoglycemia-induced brain edema in rats. PLoS ONE.

[68] Nicchia G.P., Frigeri A., Liuzzi G.M., Svelto M. (2003). Inhibition of aquaporin-4 expression in astrocytes by RNAi determines alteration in cell morphology, growth, and water transport and induces changes in ischemia-related genes. FASEB J..

[69] Belanger M., Allaman I., Magistretti P.J. (2011). Brain energy metabolism: Focus on astrocyte-neuron metabolic cooperation. Cell Metab..

[70] Felipo V. (2013). Hepatic encephalopathy: Effects of liver failure on brain function. Nat. Rev. Neurosci..

[71] Bosoi C.R., Zwingmann C., Marin H., Parent-Robitaille C., Huynh J., Tremblay M., Rose C.F. (2014). Increased brain lactate is central to the development of brain edema in rats with chronic liver disease. J. Hepatol..

[72] Rom S., Zuluaga-Ramirez V., Gajghate S., Seliga A., Winfield M., Heldt N.A., Kolpakov M.A., Bashkirova Y.V., Sabri A.K., Persidsky Y. (2019). Hyperglycemia-Driven Neuroinflammation Compromises BBB Leading to Memory Loss in Both Diabetes Mellitus (DM) Type 1 and Type 2 Mouse Models. Mol. Neurobiol..

[73] Blackburn D., Sargsyan S., Monk P.N., Shaw P.J. (2009). Astrocyte function and role in motor neuron disease: A future therapeutic target?. Glia.

[74] Coleman E., Judd R., Hoe L., Dennis J., Posner P. (2004). Effects of diabetes mellitus on astrocyte GFAP and glutamate transporters in the CNS. Glia.

[75] Sepehrinezhad A., Zarifkar A., Namvar G., Shahbazi A., Williams R. (2020). Astrocyte swelling in hepatic encephalopathy: Molecular perspective of cytotoxic edema. Metab. Brain Dis..

[76] Bak L.K., Schousboe A., Waagepetersen H.S. (2006). The glutamate/GABA-glutamine cycle: Aspects of transport, neurotransmitter homeostasis and ammonia transfer. J. Neurochem..

[77] Hakvoort T.B., He Y., Kulik W., Vermeulen J.L., Duijst S., Ruijter J.M., Runge J.H., Deutz N.E., Koehler S.E., Lamers W.H. (2017). Pivotal role of glutamine synthetase in ammonia detoxification. Hepatology.

[78] Suarez I., Bodega G., Fernandez B. (2002). Glutamine synthetase in brain: Effect of ammonia. Neurochem. Int..

[79] Tofteng F., Hauerberg J., Hansen B.A., Pedersen C.B., Jorgensen L., Larsen F.S. (2006). Persistent arterial hyperammonemia increases the concentration of glutamine and alanine in the brain and correlates with intracranial pressure in patients with fulminant hepatic failure. J. Cereb. Blood Flow Metab..

[80] Hourani B.T., Hamlin E.M., Reynolds T.B. (1971). Cerebrospinal fluid glutamine as a measure of hepatic encephalopathy. Arch. Intern. Med..

[81] Girard G., Giguere J.F., Butterworth R.F. (1993). Region-selective reductions in activities of glutamine synthetase in rat brain following portacaval anastomosis. Metab. Brain Dis..

[82] Wiegers E.C., Rooijackers H.M., van Asten J.J.A., Tack C.J., Heerschap A., de Galan B.E., van der Graaf M. (2019). Elevated brain glutamate levels in type 1 diabetes: Correlations with glycaemic control and age of disease onset but not with hypoglycaemia awareness status. Diabetologia.

[83] Li W., Roy Choudhury G., Winters A., Prah J., Lin W., Liu R., Yang S.H. (2018). Hyperglycemia Alters Astrocyte Metabolism and Inhibits Astrocyte Proliferation. Aging Dis..

[84] Azhari H., Swain M.G. (2018). Role of Peripheral Inflammation in Hepatic Encephalopathy. J. Clin. Exp. Hepatol..

[85] Akrivos J., Ravona-Springer R., Schmeidler J., LeRoith D., Heymann A., Preiss R., Hoffman H., Koifman K., Silverman J.M., Schnaider Beeri M. (2015). Glycemic control, inflammation, and cognitive function in older patients with type 2 diabetes. Int. J. Geriatr. Psychiatry.

[86] Kabba J.A., Xu Y., Christian H., Ruan W., Chenai K., Xiang Y., Zhang L., Saavedra J.M., Pang T. (2018). Microglia: Housekeeper of the Central Nervous System. Cell Mol. Neurobiol..

[87] Wright G.A., Sharifi Y., Newman T.A., Davies N., Vairappan B., Perry H.V., Jalan R. (2014). Characterisation of temporal microglia and astrocyte immune responses in bile duct-ligated rat models of cirrhosis. Liver Int..

[88] Fakhoury M. (2018). Microglia and Astrocytes in Alzheimer’s Disease: Implications for Therapy. Curr. Neuropharmacol..

[89] Wright G., Swain M., Annane D., Saliba F., Samuel D., Arroyo V., DeMorrow S., Witt A. (2016). Neuroinflammation in liver disease: Sessional talks from ISHEN. Metab. Brain Dis..

[90] Bampi S.R., Casaril A.M., Domingues M., de Andrade Lourenco D., Pesarico A.P., Vieira B., Begnini K.R., Seixas F.K., Collares T.V., Lenardao E.J. (2020). Depression-like behavior, hyperglycemia, oxidative stress, and neuroinflammation presented in diabetic mice are reversed by the administration of 1-methyl-3-(phenylselanyl)-1H-indole. J. Psychiatr. Res..

[91] Felipo V., Urios A., Montesinos E., Molina I., Garcia-Torres M.L., Civera M., Olmo J.A., Ortega J., Martinez-Valls J., Serra M.A. (2012). Contribution of hyperammonemia and inflammatory factors to cognitive impairment in minimal hepatic encephalopathy. Metab. Brain Dis..

[92] Butterworth R.F. (2011). Hepatic encephalopathy: A central neuroinflammatory disorder?. Hepatology.

[93] Deans K.A., Sattar N. (2006). “Anti-inflammatory” drugs and their effects on type 2 diabetes. Diabetes Technol. Ther..

[94] Harada S., Miyagi K., Obata T., Morimoto Y., Nakamoto K., Kim K.I., Kim S.K., Kim S.R., Tokuyama S. (2017). Influence of hyperglycemia on liver inflammatory conditions in the early phase of non-alcoholic fatty liver disease in mice. J. Pharm. Pharmacol..

[95] Palomero-Gallagher N., Zilles K. (2013). Neurotransmitter receptor alterations in hepatic encephalopathy: A review. Arch. Biochem. Biophys..

[96] Cauli O., Rodrigo R., Llansola M., Montoliu C., Monfort P., Piedrafita B., El Mlili N., Boix J., Agusti A., Felipo V. (2009). Glutamatergic and gabaergic neurotransmission and neuronal circuits in hepatic encephalopathy. Metab. Brain Dis..

[97] Son H., Jung S., Kim J.Y., Goo Y.M., Cho K.M., Lee D.H., Roh G.S., Kang S.S., Cho G.J., Choi W.S. (2015). Type 1 diabetes alters astrocytic properties related with neurotransmitter supply, causing abnormal neuronal activities. Brain Res..

[98] Monfort P., Munoz M.D., Felipo V. (2005). Chronic hyperammonemia in vivo impairs long-term potentiation in hippocampus by altering activation of cyclic GMP-dependent-protein kinase and of phosphodiesterase 5. J. Neurochem..

[99] Malone J.I., Hanna S., Saporta S., Mervis R.F., Park C.R., Chong L., Diamond D.M. (2008). Hyperglycemia not hypoglycemia alters neuronal dendrites and impairs spatial memory. Pediatr. Diabetes.

[100] Hernandez-Rabaza V., Cabrera-Pastor A., Taoro-Gonzalez L., Gonzalez-Usano A., Agusti A., Balzano T., Llansola M., Felipo V. (2016). Neuroinflammation increases GABAergic tone and impairs cognitive and motor function in hyperammonemia by increasing GAT-3 membrane expression. Reversal by sulforaphane by promoting M2 polarization of microglia. J. Neuroinflamm..

[101] Cauli O., Llansola M., Erceg S., Felipo V. (2006). Hypolocomotion in rats with chronic liver failure is due to increased glutamate and activation of metabotropic glutamate receptors in substantia nigra. J. Hepatol..

[102] van Bussel F.C., Backes W.H., Hofman P.A., Puts N.A., Edden R.A., van Boxtel M.P., Schram M.T., Stehouwer C.D., Wildberger J.E., Jansen J.F. (2016). Increased GABA concentrations in type 2 diabetes mellitus are related to lower cognitive functioning. Medicine (Baltimore).

[103] Wu H., Denna T.H., Storkersen J.N., Gerriets V.A. (2019). Beyond a neurotransmitter: The role of serotonin in inflammation and immunity. Pharmacol. Res..

[104] Wise R.A. (2004). Dopamine, learning and motivation. Nat. Rev. Neurosci..

[105] Chojnacki C., Walecka-Kapica E., Stepien A., Pawlowicz M., Wachowska-Kelly P., Chojnacki J. (2013). Serum and ascitic fluid serotonin levels and 5-hydroxyindoleacetic acid urine excretion in the liver of cirrhotic patients with encephalopathy. Adv. Med. Sci..

[106] Borg J., Warter J.M., Schlienger J.L., Imler M., Marescaux C., Mack G. (1982). Neurotransmitter modifications in human cerebrospinal fluid and serum during hepatic encephalopathy. J. Neurol. Sci..

[107] Zhuge W., Wen F., Ni Z., Zheng Z., Zhu X., Lin J., Wang J., Zhuge Q., Ding S. (2019). Dopamine Burden Triggers Cholesterol Overload Following Disruption of Synaptogenesis in Minimal Hepatic Encephalopathy. Neuroscience.

[108] Dhanda S., Sandhir R. (2015). Role of dopaminergic and serotonergic neurotransmitters in behavioral alterations observed in rodent model of hepatic encephalopathy. Behav. Brain Res..

[109] Parashar A., Mehta V., Malairaman U. (2018). Type 2 Diabetes Mellitus Is Associated with Social Recognition Memory Deficit and Altered Dopaminergic Neurotransmission in the Amygdala. Ann. Neurosci..

[110] Kleinridders A., Cai W., Cappellucci L., Ghazarian A., Collins W.R., Vienberg S.G., Pothos E.N., Kahn C.R. (2015). Insulin resistance in brain alters dopamine turnover and causes behavioral disorders. Proc. Natl. Acad. Sci. USA.

[111] Borodovitsyna O., Flamini M., Chandler D. (2017). Noradrenergic Modulation of Cognition in Health and Disease. Neural Plast.

[112] Kawai H., Ishibashi T., Kudo N., Kawashima Y., Mitsumoto A. (2012). Behavioral and biochemical characterization of rats treated chronically with thioacetamide: Proposal of an animal model for hepatic encephalopathy associated with cirrhosis. J. Toxicol. Sci..

[113] Garris D.R. (1990). Age- and diabetes-associated alterations in regional brain norepinephrine concentrations and adrenergic receptor populations in C57BL/KsJ mice. Brain Res. Dev. Brain Res..

[114] Rahn K.A., Slusher B.S., Kaplin A.I. (2012). Glutamate in CNS neurodegeneration and cognition and its regulation by GCPII inhibition. Curr. Med. Chem..

[115] Monfort P., Erceg S., Piedrafita B., Llansola M., Felipo V. (2007). Chronic liver failure in rats impairs glutamatergic synaptic transmission and long-term potentiation in hippocampus and learning ability. Eur. J. Neurosci..

[116] Llansola M., Montoliu C., Cauli O., Hernandez-Rabaza V., Agusti A., Cabrera-Pastor A., Gimenez-Garzo C., Gonzalez-Usano A., Felipo V. (2013). Chronic hyperammonemia, glutamatergic neurotransmission and neurological alterations. Metab. Brain Dis..

[117] Lyoo I.K., Yoon S.J., Musen G., Simonson D.C., Weinger K., Bolo N., Ryan C.M., Kim J.E., Renshaw P.F., Jacobson A.M. (2009). Altered prefrontal glutamate-glutamine-gamma-aminobutyric acid levels and relation to low cognitive performance and depressive symptoms in type 1 diabetes mellitus. Arch. Gen. Psychiatry.

[118] Bartus R.T., Dean R.L., Beer B., Lippa A.S. (1982). The cholinergic hypothesis of geriatric memory dysfunction. Science.

[119] Mooradian A.D. (1987). Blood-brain barrier choline transport is reduced in diabetic rats. Diabetes.

[120] Binesh N., Huda A., Thomas M.A., Wyckoff N., Bugbee M., Han S., Rasgon N., Davanzo P., Sayre J., Guze B. (2006). Hepatic encephalopathy: A neurochemical, neuroanatomical, and neuropsychological study. J. Appl. Clin. Med. Phys..

[121] Romero-Gomez M., Jover M., Del Campo J.A., Royo J.L., Hoyas E., Galan J.J., Montoliu C., Baccaro E., Guevara M., Cordoba J. (2010). Variations in the promoter region of the glutaminase gene and the development of hepatic encephalopathy in patients with cirrhosis: A cohort study. Ann. Intern. Med..

[122] Dongiovanni P., Anstee Q.M., Valenti L. (2013). Genetic predisposition in NAFLD and NASH: Impact on severity of liver disease and response to treatment. Curr. Pharm. Des..

[123] Sirdah M.M., Reading N.S. (2020). Genetic predisposition in type 2 diabetes: A promising approach toward a personalized management of diabetes. Clin. Genet..

[124] Smith E.M., Watford M. (1990). Molecular cloning of a cDNA for rat hepatic glutaminase. Sequence similarity to kidney-type glutaminase. J. Biol. Chem..

[125] De la Monte S.M., Tong M. (2014). Brain metabolic dysfunction at the core of Alzheimer’s disease. Biochem. Pharmacol..

[126] Seaquist E.R., Damberg G.S., Tkac I., Gruetter R. (2001). The effect of insulin on in vivo cerebral glucose concentrations and rates of glucose transport/metabolism in humans. Diabetes.

[127] Ribeiro I.M., Ferreira-Neto H.C., Antunes V.R. (2015). Subdiaphragmatic vagus nerve activity and hepatic venous glucose are differentially regulated by the central actions of insulin in Wistar and SHR. Physiol. Rep..

[128] Plum L., Belgardt B.F., Bruning J.C. (2006). Central insulin action in energy and glucose homeostasis. J. Clin. Investig..

[129] Cholerton B., Baker L.D., Craft S. (2013). Insulin, cognition, and dementia. Eur. J. Pharmacol..

[130] De la Monte S.M., Wands J.R. (2008). Alzheimer’s disease is type 3 diabetes-evidence reviewed. J. Diabetes Sci. Technol..

[131] Morris J.K., Vidoni E.D., Honea R.A., Burns J.M., Alzheimer’s Disease Neuroimaging Initiative (2014). Impaired glycemia increases disease progression in mild cognitive impairment. Neurobiol. Aging.

[132] Han H.S., Kang G., Kim J.S., Choi B.H., Koo S.H. (2016). Regulation of glucose metabolism from a liver-centric perspective. Exp. Mol. Med..

[133] David-Silva A., Esteves J.V., Morais M., Freitas H.S., Zorn T.M., Correa-Giannella M.L., Machado U.F. (2020). Dual SGLT1/SGLT2 Inhibitor Phlorizin Ameliorates Non-Alcoholic Fatty Liver Disease and Hepatic Glucose Production in Type 2 Diabetic Mice. Diabetes Metab. Syndr. Obes..

[134] Li X., Jiao Y., Xing Y., Gao P. (2019). Diabetes Mellitus and Risk of Hepatic Fibrosis/Cirrhosis. BioMed Res. Int..

[135] Deng D., Yan N. (2016). GLUT, SGLT, and SWEET: Structural and mechanistic investigations of the glucose transporters. Protein Sci..

[136] Pardridge W.M., Triguero D., Farrell C.R. (1990). Downregulation of blood-brain barrier glucose transporter in experimental diabetes. Diabetes.

[137] Vannucci S.J., Gibbs E.M., Simpson I.A. (1997). Glucose utilization and glucose transporter proteins GLUT-1 and GLUT-3 in brains of diabetic (db/db) mice. Am. J. Physiol..

[138] Belanger M., Desjardins P., Chatauret N., Butterworth R.F. (2006). Selectively increased expression of the astrocytic/endothelial glucose transporter protein GLUT1 in acute liver failure. Glia.

[139] Wanner C., Marx N. (2018). SGLT2 inhibitors: The future for treatment of type 2 diabetes mellitus and other chronic diseases. Diabetologia.

[140] Zhang W., Ning N., Li X., Li M., Duan X., Guo Y., Dang Y., Li Y., Gao J., Ye J. (2019). Impaired brain glucose metabolism in cirrhosis without overt hepatic encephalopathy: A retrospective 18F-FDG PET/CT study. Neuroreport.

[141] Poh Z., Chang P.E. (2012). A current review of the diagnostic and treatment strategies of hepatic encephalopathy. Int. J. Hepatol..

[142] Hawkins R.A., Jessy J., Mans A.M., Chedid A., DeJoseph M.R. (1994). Neomycin reduces the intestinal production of ammonia from glutamine. Adv. Exp. Med. Biol..

[143] Ndraha S., Hasan I., Simadibrata M. (2011). The effect of L-ornithine L-aspartate and branch chain amino acids on encephalopathy and nutritional status in liver cirrhosis with malnutrition. Acta Med. Indones.

[144] Hopp A.E., Dirks M., Petrusch C., Goldbecker A., Tryc A.B., Barg-Hock H., Strassburg C., Klempnauer J., Weissenborn K., Pflugrad H. (2019). Hepatic Encephalopathy Is Reversible in the Long Term After Liver Transplantation. Liver Transpl..

